# Acute kidney tubular injury after ingestion of red yeast rice supplement

**DOI:** 10.1093/ckj/sfae151

**Published:** 2024-05-16

**Authors:** Reina Miyazaki, Yasuhito Takahashi, Tetsuya Kawamura, Hiroyuki Ueda, Nobuo Tsuboi, Takashi Yokoo

**Affiliations:** Division of Nephrology and Hypertension, Department of Internal Medicine, Jikei University School of Medicine, Tokyo, Japan; Division of Nephrology, Department of Internal Medicine, Fuji City General Hospital, Shizuoka, Japan; Division of Nephrology and Hypertension, Department of Internal Medicine, Jikei University School of Medicine, Tokyo, Japan; Division of Nephrology, Department of Internal Medicine, Fuji City General Hospital, Shizuoka, Japan; Division of Nephrology and Hypertension, Department of Internal Medicine, Jikei University School of Medicine, Tokyo, Japan; Division of Nephrology, Department of Internal Medicine, Fuji City General Hospital, Shizuoka, Japan; Division of Nephrology and Hypertension, Department of Internal Medicine, Jikei University School of Medicine, Tokyo, Japan; Division of Nephrology and Hypertension, Department of Internal Medicine, Jikei University School of Medicine, Tokyo, Japan; Division of Nephrology and Hypertension, Department of Internal Medicine, Jikei University School of Medicine, Tokyo, Japan

**Keywords:** acute tubular necrosis, kidney biopsy, mycotoxin, red yeast rice, supplement

## Abstract

A 47-year-old woman developed severe kidney dysfunction after taking a lipid-lowering supplement, Red Yeast Rice Cholestehelp, for approximately 7 months. The patient developed sudden nausea and had an elevated serum creatinine level of 4.26 mg/dL. A kidney biopsy showed findings consistent with acute tubular necrosis. Kidney dysfunction improved with discontinuation of supplementation, and corticosteroid therapy. Similar kidney involvement has been reported, raising concerns regarding supplements in Japan. An investigation of the nephrotoxic ingredients in the same product batches is currently underway. This report underscores the need for public awareness and warnings of health risk concerns associated with unregulated supplements.

## INTRODUCTION

Red yeast is widely used as a food coloring, traditional Chinese medicine and dietary supplement, especially in Asia. By 17 April 2024, 236 cases of health hazards requiring hospitalization, including serious kidney dysfunction, suspected to be related to red yeast rice supplements had been identified in Japan, leading to an urgent recall of the supplements [1].

We herein report a case of severe kidney dysfunction that may have been associated with supplements containing red yeast.

## CASE REPORT

The patient was a 47-year-old woman diagnosed with dyslipidemia by her physician. She started taking the supplement “Red Yeast Rice Cholestehelp” (Kobayashi Pharmaceutical Co., Ltd) to treat dyslipidemia at her own discretion, taking 0–3 tablets per day for a total of 4–5 packets (240–300 tablets), for approximately 9 months. Before starting the supplement, her serum creatinine level was 1.09 mg/dL and estimated glomerular filtration rate was 44 mL/min/1.73 m^2^, with normal urinalysis findings. Five days prior to admission, she visited her doctor because of nausea. From that day onward, she stopped taking the red yeast rice supplement. The patient did not complain of any muscle or joint pain, or urological manifestations including oliguria. Blood tests revealed an elevated serum creatinine level of 4.26 mg/dL, and urinary tests showed proteinuria, granular casts, tubular epithelial cells and glycosuria. She had no recent history of medications, except for the red yeast rice supplement, prior to the onset of acute kidney injury (AKI).

On admission, blood pressure was 144/88 mmHg, and body temperature was 36.6°C. Physical examination findings were uneventful. Laboratory findings on admission are shown in Supplementary data, Fig. S1. Urinalysis showed high levels of urinary β2-microglobulin (109.677 μg/L) and N-acetyl-β-d-glucosaminidase (16.6 U/L). Serum potassium (3.6 mEq/L) and urate (2.5 mg/dL) levels were low. Computed tomography revealed a preserved bilateral kidney morphology. A kidney biopsy was performed to investigate the kidney dysfunction etiology. Tubular dilatation, epithelial desquamation, thinning and hyaline casts, but neither diffuse interstitial infiltration nor tubulitis was observed, suggestive of acute tubular necrosis.

**Figure 1:**
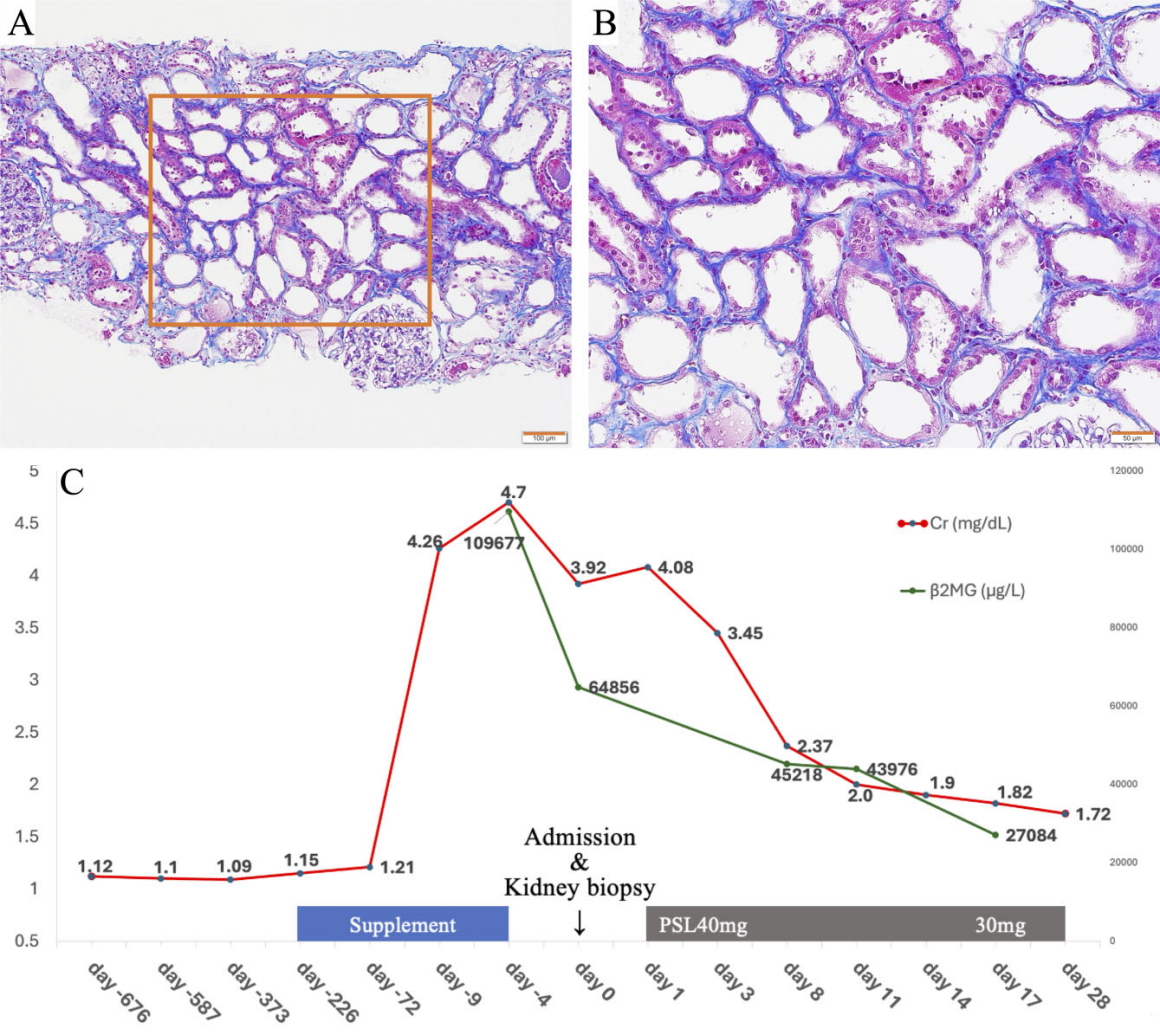
Kidney biopsy findings and clinical course. (**A, B**) The tubular lumen was moderately enlarged, and the tubular epithelium was markedly flattened. ×100, ×200, Masson's trichrome staining. Of the 26 glomeruli identified, 14 were globally sclerotic, but the remaining glomeruli showed no abnormalities other than mild glomerular collapse. The arteries showed moderate arteriosclerosis. Immunostaining and electron microscopy did not reveal any significant findings. (**C**) Kidney dysfunction steadily improved after discontinuation of the supplementation and corticosteroid therapy. PSL, predonisolone; β2MG, beta-2 microglobulin (μg/L).

Physical and laboratory examinations on admission did not reveal any obvious etiology associated with AKI. Under the assumption of tubulointerstitial nephritis due to the red yeast supplement, corticosteroid therapy was administered with a starting dose of 40 mg of oral prednisone (0.8 mg/kg) per day. As the kidney function improved over time and kidney biopsy revealed tubular necrosis, corticosteroids were tapered, and her serum creatinine improved to 1.72 mg/dL approximately 4 weeks after admission (Figure 1).

## DISCUSSION

In 1997, Chinese researchers reported that red yeast rice preparations had lipid-lowering properties [2], and many pharmaceutical companies marketed these products. In 2007, the US Food and Drug Administration warned consumers to avoid red yeast rice products because they contained nephrotoxic ingredients, including citrinin, a type of mycotoxin [3, 4]. However, Japanese red yeast rice, mainly fermented by *Monascus pilosus*, has been proven to be safe because it is genetically incapable of producing citrinin [5].

The substance responsible for the AKI in this case is unknown. Unlike previously reported cases of AKI related to red yeast rice Supplemental reference S1–S3, rhabdomyolysis was not identified. Consistent with the laboratory findings indicative of Fanconi syndrome, a kidney biopsy histopathology suggested tubular necrosis, rather than tubulointerstitial nephritis. Currently, there are an increasing number of reports of severe kidney dysfunction, similar to our case, in patients who have taken red yeast rice supplements in Japan [1]. Each package of the red yeast rice supplement contained 60 tablets for 20 days (3 tablets per day). Since our patient had ingested 0–3 tablets per day for 7 months and AKI was not evident in the early stages of ingestion, we suspected an association with a recently ingested batch or a lot of red yeast rice supplement. According to the company that manufactures the product, patients suffering from kidney dysfunction have ingested products made from the same lot of raw materials, and an analysis has suggested that the red yeast rice supplement may contain nephrotoxic ingredients other than citrinin [1].

In the present case, tubulointerstitial nephritis was first postulated as the pathogenesis of AKI associated with the red rice yeast supplement, and corticosteroid therapy was introduced; however, it is difficult to determine the effectiveness of corticosteroids for this condition, because the patient might have recovered from kidney dysfunction through a natural course. This patient had mild kidney dysfunction prior to taking the supplement. Considering that kidney dysfunction was evident only in the minority of patients who took the red yeast rice supplement, the underlying chronic kidney disease may have increased susceptibility to AKI in this condition.

## Supplementary Material

sfae151_Supplemental_File

## Data Availability

The data underlying this article are available in the article and in its online Supplementary data.
