# Supplementary material for: Acute kidney tubular injury after ingestion of red yeast rice supplement
Source: Clin Kidney J. 2024 May 16;17(6):sfae151. doi: 10.1093/ckj/sfae151 (PMC11153872; doi:10.1093/ckj/sfae151)
Supplement: sfae151_Supplemental_File [file sfae151_supplemental_file.pdf]

Supplementary Materials

Acute tubular injury after ingestion of red yeast rice supplement

Miyazaki R, Takahashi Y, Kawamura T, Ueda H, Tsuboi N and Yokoo T

Table of Contents

- Supplementary Table S1. Laboratory data on admission
- Supplementary References

Supplemental Table

Table S1. Laboratory data on admission

	Reference range	Results
Blood cell count		
White blood cell count (/μL)	3,300-8,600	8,700
Eosinophil count (/μL)	0-300	200
Red blood cell count (/μL)	3,860,000-4920000	4,830,000
Hemoglobin (g/dL)	11.6-14.8	13.7
Platelet count (/μL)	158,000-348000	268,000
Serum biochemistry		
Total protein(g/dL)	6.6–8.1	7.7
Albumin (g/dL)	4.1–5.1	4.6
Total bilirubin (mg/dL)	0.4–1.5	0.4
Alanine aminotransferase (U/L)	13–30	17
Aspartate aminotransferase (U/L)	7–23	15
Alkaline phosphatase (U/L)	106–322	88
Creatine kinase (U/L)	41-153	79
Lactate dehydrogenase (U/L)	124-222	316
Creatinine (mg/dL)	0.46–0.79	4.70
C-reactive protein (mg/L)	>0.14	0.09
Sodium (mEq/L)	138-145	138
Potassium (mEq/L)	3.6-4.8	3.6
Chloride (mEq/L)	101-108	111
Urine acid (mg/dL)	2.6-5.5	2.5
Calcium (mg/dL)	8.8-10.1	9.3
Phosphorus (mg/dL)	2.7-4.6	3.7
Beta-2 microglobulin (mg/L)	1.0-1.9	4.85
Hemoglobin A1c (%)	4.9-6.0	5.9
Glucose (mg/dL)	73-109	85
Triglycerides (mg/dL)	30-117	84
Total cholesterol (mg/dL)	142-248	235
low-density lipoprotein cholesterol (mg/dL)	65-163	147
Immunoglobulin G (mg/dL)	870–1,700	1312
Immunoglobulin G4 (mg/dL)	11-121	46
Immunoglobulin A (mg/dL)	110–410	236
Immunoglobulin M (mg/dL)	35–220	150
Complement component 3 (mg/dL)	65–135	115.3
Complement component 4 (mg/dL)	13–35	38.9
CH50 (U/mL)	30–50	67.7
Antinuclear antibody	0–39	<40
Rheumatoid factor	0–15	2
Antistreptolysin O (U/mL)	<160	163
Serum cryoglobulin	Negative	Negative
Monoclonal protein	Negative	Negative
Proteinase 3-anti-neutrophil cytoplasmic antibodies	Negative	Negative
Myeloperoxidase-anti-neutrophil cytoplasmic antibodies	Negative	Negative
Anti-glomerular basement membrane antibodies	Negative	Negative

Hepatitis B surface antigen	Negative	Negative
Hepatitis B surface antibody	Negative	Negative
Hepatitis B core antibody	Negative	Negative
Hepatitis C virus antibody	Negative	Negative
Urinalysis		
Specific gravity		1.024
pH		6.0
Glucose		4+
Protein		3+
Hematuria (/high power field)		5-9
Granular cast (/high power field)		10-19
Tubular epithelium (/5high power field)		1
Urinary beta-2 microglobulin (μg/L)		109,677
N-acetyl-beta-D-glucosaminidase (U/L)		16.6
24-hour Urinalysis		
Protein (mg/day)		1,384
Creatinine clearance (mL/min)		15

Supplemental References

1. Rhabdomyolysis linked to Chinese red yeast rice. Prescrire Int. 2008; 17 (94): 64.

2. Peterslund P, Christensen HD, Urbahnke J, Cappeln AV. [Red yeast rice as the presumed cause of acute kidney and liver failure]. Ugeskr Laeger. 2019; 30,181 (40): V02190107. Danish. PMID: 31566178.

3. Prasad GV, Wong T, Meliton G, Bhaloo S. Rhabdomyolysis due to red yeast rice (Monascus purpureus) in a renal transplant recipient. Transplantation. 2002; 27,74 (8): 1200-1.
